# Supplementary material for: The interactome of intact mitochondria by cross-linking mass spectrometry provides evidence for coexisting respiratory supercomplexes
Source: Mol Cell Proteomics. 2017 Dec 8;17(2):216–32. doi: 10.1074/mcp.RA117.000470 (PMC5795388; doi:10.1074/mcp.RA117.000470)
Supplement: Supplemental Data [file supp_RA117.000470_133922_0_supp_23978_fzffwf.zip › spectra_annotation/mito_spectra_annotation/394-1-112-1-12-1.pdf]

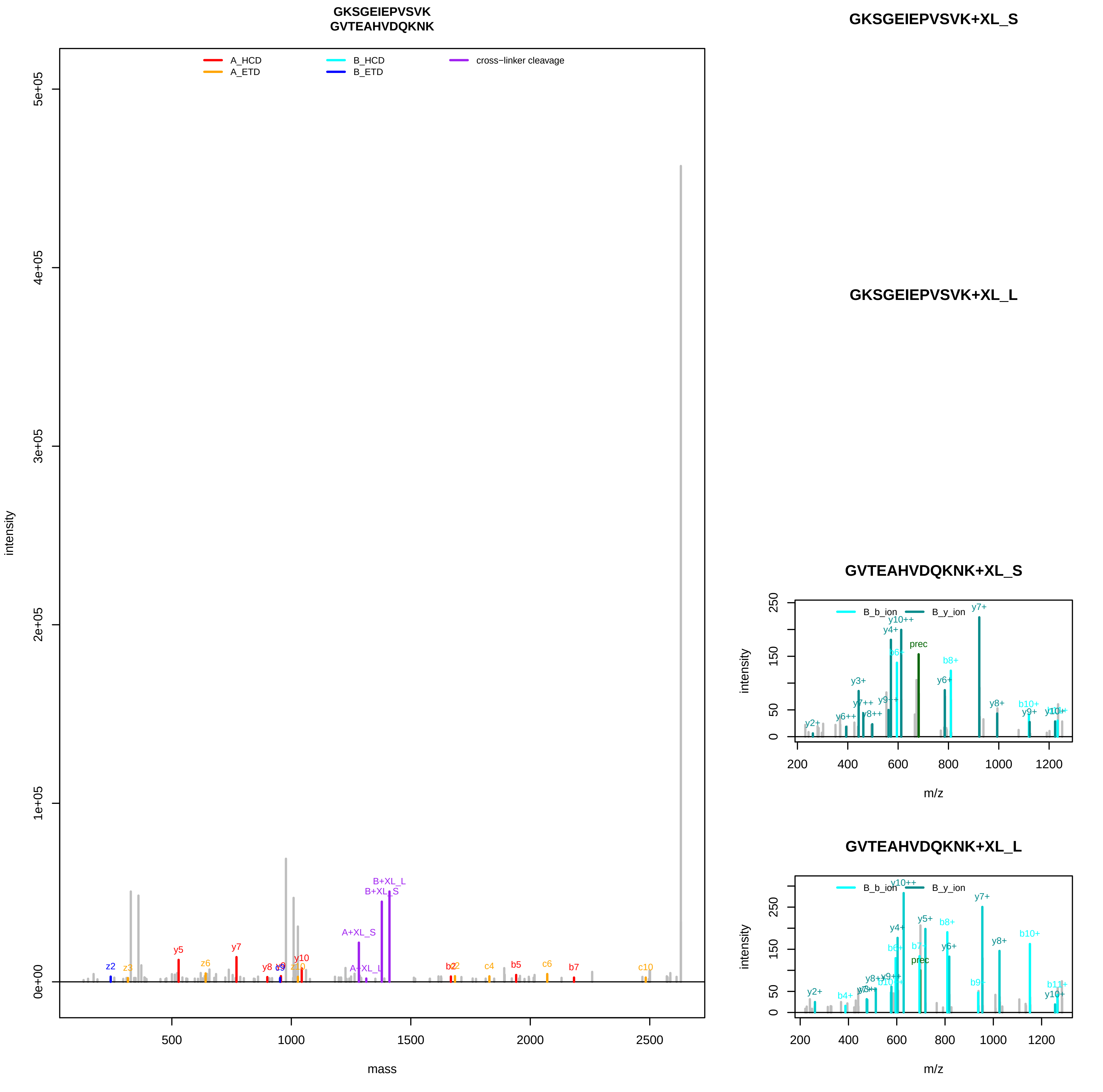

GKSGEIEPVSVK
GVTEAHVDQKNK
A_HCD
A_ETD
B_HCD
B_ETD
cross−linker cleavage
intensity
mass
0e+00
1e+05
2e+05
3e+05
4e+05
5e+05
500
1000
1500
2000
2500
z2
z3
y5
z6
y7
y8
y9
c9
z10
y10
A+XL_S
A+XL_L
B+XL_S
B+XL_L
b2
c2
c4
b5
c6
b7
c10
GKSGEIEPVSVK+XL_S
GKSGEIEPVSVK+XL_L
GVTEAHVDQKNK+XL_S
B_b_ion
B_y_ion
m/z
y2+
y3+
y4+
y6++
y7++
y8++
y9++
y10++
b6+
prec
b8+
y6+
y7+
y8+
b10+
y9+
y10+
GVTEAHVDQKNK+XL_L
b4+
y5+
b7+
b9+
b11+
